# Case report: Reversible restricted diffusion and cytotoxic edema in the perilesional zone following continuous partial seizures

**DOI:** 10.4103/0971-3026.41833

**Published:** 2008-08

**Authors:** MK Goyal, S Sinha, S Ravishankar, JJ Shivshankar

**Affiliations:** Department of Neurology, National Institute of Mental Health and Neurosciences, Bangalore, India; 1 Department of Neuroradiology, National Institute of Mental Health and Neurosciences, Bangalore, India

**Keywords:** Partial seizures, cytotoxic edema, diffusion MRI

MRI reveals restricted diffusion on diffusion-weighted imaging (DWI) and apparent diffusion coefficient (ADC) in the region of cytotoxic edema, while no such restriction is observed in vasogenic or perilesional edema. We present a case where the patient manifested with status epilepticus (SE) due to a granuloma, possibly neurocysticercosis, with reversible restricted diffusion and cytotoxic edema on DWI and ADC in the perilesional zone of altered signal intensity.

## Case History

**Figure 1 (A-D):**
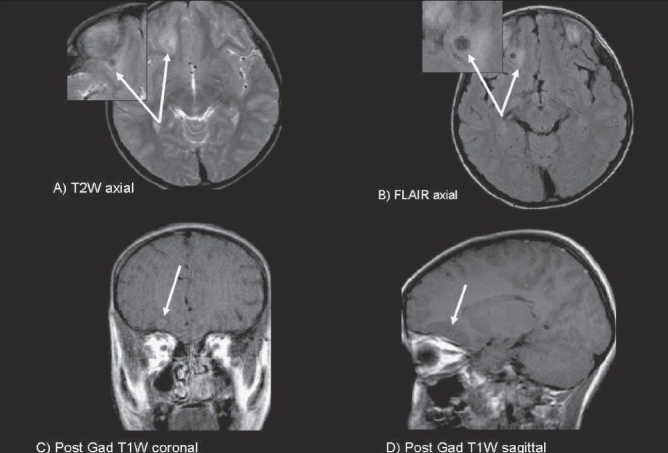
T2W [A] and FLAIR [B] images show hyperintense signal (white arrows) in the right basifrontal region with a central focal area of hypointensity. Postcontrast (gadolinium) axial (C) and sagittal (D) images reveal a ring-enhancing lesion with perilesional edema (white arrow). These findings were suggestive neurocysticercosis

**Figure 2 (Aa-Dd):**
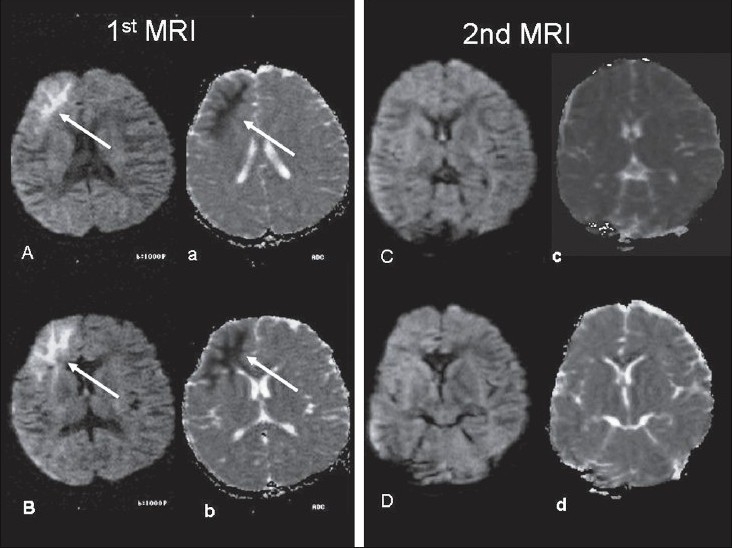
First MRI (A, a; B, b): DWI (A, B) images show hyperintensity (white arrow) involving the grey and white matter in the right basifrontal region with ADC images (a, b) showing hypointensity (white arrow) in the same region, suggesting cytotoxic edema and restricted diffusion. Repeat MRI after 4 days with DWI (C, D) and ADC (c, d) images, shows resolution of the restricted diffusion

A 10-year-old boy presented with recurrent left partial motor seizures (20-24/day) for 6 days with secondary generalization and unconsciousness. He had only received 50 mg of oral phenytoin twice a day prior to admission. There was no other significant history. The vital signs were stable and he had crackles in the chest. The Glasgow coma score was 6/15 (E2, M3, V1). There was no lateralizing sign. The seizures were brought under control with intravenous lorazepam, phenytoin, and valproic acid in recommended dosages 45 min after admission. Hemogram and serum biochemical parameters, including glucose, liver and renal function tests, and electrolytes, were normal. MRI was performed on 15^th^ May 2006 within 24 h of cessation of SE and showed a right frontal ring-enhancing lesion (8 mm in diameter), with perilesional hyperintensity on T2W, FLAIR, and PDW sequences [Figure 1 A-B], findings that were suggestive of neurocysticercosis. On b = 1000s/mm DWI [Figure 2], the zone surrounding the granuloma was hyperintense and on the corresponding ADC maps there was hypointensity, suggesting restricted diffusion, i.e., cytotoxicity. The ADC value in the perilesional zone was 23.6 × 10^−3^ mm^2^ per second on the side of the lesion as compared to 83.6 × 10^−3^ mm^2^ per second in the corresponding region on the healthy side, suggesting a percentage decrease of 71.8% in the ADC coefficient. The area measured contained white matter. A repeat DWI study after 96 h revealed no restriction and the ADC value in the perilesional zone was 71 × 10^−3^ mm^2^ per second on the side of the lesion as compared to 76 × 10^−3^ mm^2^ per second in the corresponding region on the healthy side. However, there was no further serial MRI study. EEG immediately after control of seizures showed diffuse theta slowing of background activity with intermittent, frontally dominant, delta waves, which was more from the right side.

## Discussion

Conventionally, the edema in the vicinity of brain tumors, cerebral abscesses, and granulomas is vasogenic, because there is disruption of the blood-brain barrier. It is characterized by variable signal intensity on DWI and high ADC values. Symptomatic SE secondary to granulomas is characterized by surrounding vasogenic edema and this is often noted following a seizure. By contrast, cytotoxic edema is characterized by high signal intensity on DWI with low ADC values (hypointense signal on ADC). In this patient, MRI findings were suggestive of neurocysticercosis. The DWI revealed hyperintense signal change in the perilesional zone, which was hypointense on ADC, suggesting restricted diffusion (cytotoxic edema). A repeat study with DWI and ADC 4 days later revealed resolution of the cytotoxic edema. To the best of our knowledge, this is a novel observation. Evidence of cytotoxic edema secondary to epileptic activity itself has been reported by several authors,[1,2] along with reduced ADC values in the perilesional region of a brain tumor.[3] Farina *et al.*[4] studied five children, aged 17 months to 7 years, with new-onset prolonged seizures and showed MRI findings of increased signal on T2W images and DWI throughout the affected hippocampus, indicating restricted diffusion. All the five patients showed hippocampal atrophy on follow-up after 2-18 months. In four patients, serial evaluation revealed increased ADC values. The authors postulated that DWI might represent a useful adjunct to conventional MRI for identifying acute injury to the hippocampus, which later might evolve into sclerosis.

Lansberg *et al.*[2] reported the utility of DWI in acute cerebral ischemia. The authors suggested that the depletion of metabolic substrates leads to failure of the Na^+^-K^+^-ATPase pump in the acute stage of cerebral ischemia and subsequently leads to cytotoxic edema, thereby creating an environment that hinders water diffusion. In ischemic brain tissue, DWI intensity is high and the ADC, a measure of freedom of water diffusion, is low.[2,5]

In experimental animals with kainate-induced SE, decreased ADC values similar to that seen in acute cerebral ischemia have been documented.[6] These changes in diffusion can be explained in the light of experimental histological studies associated with focal SE, which show swelling of astrocytes and dendrites in the affected brain of experimental subjects with SE 2-24 h after systemic injection of kainic acid.[7] The initial decrease in ADC in partial SE could be explained by the appearance of cytotoxic edema in the early stages of seizure activity. A failure of the Na^+^-K^+^-ATPase pump due to depletion of metabolic substrates, may lead to a disturbance of membrane ion homeostasis, with influx of Na^+^ into the cell,[1] which has been shown to occur in the piriform cortex of rats during focal SE.[8] The increase in ADC 72 h after the onset of seizures is probably due to the loss of diffusion barriers following cell death and neutrophil fragmentation. Similar observations have been made in acute cerebral ischemia, with an initial decrease in diffusion, reflecting cytotoxic edema, and subsequent increased diffusion after cell fragmentation.[9] In the present study, there was disappearance of these DWI and ADC abnormalities within 4 days, indicating that cytotoxic edema might resolve. Similar evidence of histological reversibility in rats[10] and reversibility on imaging[8] have been described earlier.

The finding of restricted diffusion surrounding a lesion is an interesting observation and suggests cytotoxic edema. Resolution of cytotoxicity is of great interest because it offers scope for research in epilepsy and ischemia.
